# Bis(1,3,4-thia­diazol-2-yl) disulfide

**DOI:** 10.1107/S1600536809036782

**Published:** 2009-09-16

**Authors:** Pu-Zhou Hu, Yong-Hua Zhang, Jian-Ge Wang, Jian-Hua Qin, Bang-Tun Zhao

**Affiliations:** a College of Chemistry and Chemical Engineering, Luoyang Normal University, Luoyang 471022, People’s Republic of China.

## Abstract

The title compound, C_4_H_2_N_4_S_4_, lies about a twofold rotation axis situated at the mid-point of the central S—S bond. Each of two thia­diazole rings is essentially planar, with an rms deviation for the unique thia­diazole ring plane of 0.0019 (7) Å. C—H⋯N hydrogen bonds link adjacent mol­ecules, forming zigzag chains along the *c* axis. In addition, these chains are connected by inter­molecular S⋯S inter­actions [S⋯S = 3.5153 (11) Å] , forming corrugated sheets, and further fabricate a three-dimensional supra­molecular structure by inter­molecular N⋯S contacts [S⋯N = 3.1941 (17) Å].

## Related literature

For potential applications of thia­diazo­les, see: Coyanis *et al.* (2002 ▶); Wang & Cao (2005 ▶). For their use as ligands in transition-metal coordination chemistry, see: Huang *et al.* (2004 ▶); Zheng *et al.* (2005 ▶). For the structure of bis­(2-methyl-1,3,4-thia­diazol­yl)-5,5′-disulfide, see: Hipler *et al.* (2003 ▶).
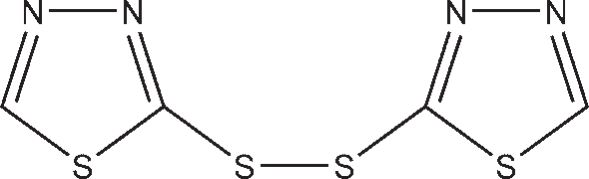


## Experimental

### 

#### Crystal data

C_4_H_2_N_4_S_4_
*M* *_r_* = 234.34
Monoclinic, 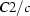

*a* = 9.706 (2) Å
*b* = 4.8980 (12) Å
*c* = 18.008 (5) Å
β = 100.074 (3)°
*V* = 842.9 (4) Å^3^
*Z* = 4
Mo *K*α radiation
μ = 1.07 mm^−1^
*T* = 291 K
0.29 × 0.20 × 0.11 mm

#### Data collection

Bruker SMART CCD area detector diffractometer
Absorption correction: multi-scan (*SADABS*; Bruker, 1997 ▶) *T*_min_ = 0.746, *T*_max_ = 0.889
2915 measured reflections
783 independent reflections
727 reflections with *I* > 2σ(*I*)
*R*_int_ = 0.017

#### Refinement

*R*[*F* ^2^ > 2σ(*F* ^2^)] = 0.021
*wR*(*F* ^2^) = 0.055
*S* = 1.11
783 reflections
55 parameters
H-atom parameters constrained
Δρ_max_ = 0.17 e Å^−3^
Δρ_min_ = −0.26 e Å^−3^

### 

Data collection: *SMART* (Bruker, 1997 ▶); cell refinement: *SAINT* (Bruker, 1997 ▶); data reduction: *SAINT*; program(s) used to solve structure: *SHELXS97* (Sheldrick, 2008 ▶); program(s) used to refine structure: *SHELXL97* (Sheldrick, 2008 ▶); molecular graphics: *SHELXTL*; software used to prepare material for publication: *SHELXTL*.

## Supplementary Material

Crystal structure: contains datablocks I, global. DOI: 10.1107/S1600536809036782/sj2643sup1.cif

Structure factors: contains datablocks I. DOI: 10.1107/S1600536809036782/sj2643Isup2.hkl

Additional supplementary materials:  crystallographic information; 3D view; checkCIF report

## Figures and Tables

**Table 1:** Hydrogen-bond geometry (Å, °)

*D*—H⋯*A*	*D*—H	H⋯*A*	*D*⋯*A*	*D*—H⋯*A*
C2—H2⋯N2^i^	0.93	2.52	3.249 (2)	136

Symmetry code: (i) 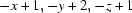.

**Fig. 1.**
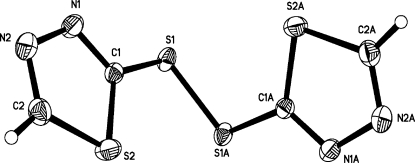
View of (I) with the atom-numbering scheme. Displacement ellipsoids are drawn at the 30% probability level.

**Fig. 2.**
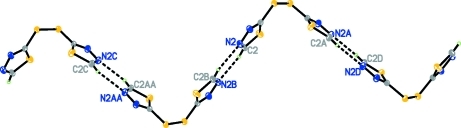
The 1-dimensional zigzag chain formed by intermolecular C-H···N interactions, shown as dashed lines.

**Fig. 3.**
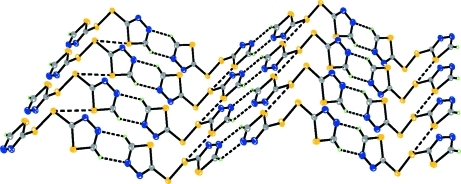
Corrugated sheets formed by intermolecular C-H···N and S···S interactions, shown as dashed lines.

**Fig. 4.**
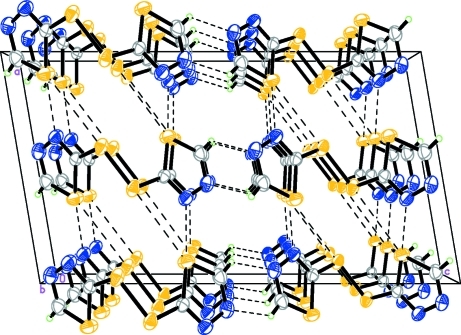
The 3-dimensional network structure formed by intermolecular C–H···N, S···S and N···S interactions, shown as dashed lines.

## supplementary crystallographic information

### Comment

Thiadiazoles have attracted increasing attention because of their potential applications in pharmaceutical, agricultural, industrial, coordination and polymer chemistry (Coyanis *et al.*, 2002, Wang & Cao, 2005). Ligands involving thiadiazole group have also shown interesting coordination chemistry with transition metal ions (Huang *et al.*, 2004; Zheng *et al.*, 2005). Exploring the applications of thiadiazole derivatives as ligands for metal complexation, we report here the synthesis and structure of bis(1,3,4-thiadiazolyl)5,5'-disulfide (I), a new and potentially multi-functional ligand (Fig. 1).

The title compound,C_4_H_2_N_4_S_4_, lies about a twofold rotation axis situated at the mid-point of the central S–S bond. Each of the thiadiazole rings is essentially planar, with an rms deviation for the unique thiadiazole ring plane of 0.0019 (7)Å.  The dihedral angle and centroid-centroid distance between the two thiadaizole rings are 86.64 (44)° and 5.25 (14) Å, respectively.  The N1-C1 and N2-C2 bond lengths, 1.298 (2) Å and 1.290 (2) Å, respectively, indicate significant double bond character, which is very similar to the structure of bis(2-methyl-1,3,4-thiadiazolyl)-5,5'-disulfide (Hipler,*et al.*, 2003).

In the crystal structure, molecules of (I) form 1-dimensional zigzag chains by way of weak intermolecular C-H···N hydrogen bonds along the *c* axis (Fig.3). In addition, these chains are linked by intermolecular S···S interactions [S2···S1 =  3.5153 (11) Å] to form corrugated sheets (Fig. 3). Further intermolecular N···S interactions (S2···N1 = 3.1941 (17) Å] generate a 3-dimensional supramolecular network structure (Fig. 4).

### Experimental

The title compound was prepared by adding hydrogen peroxide (30%, 10.4 mL) drop-wise to a solution of 2-mercapto-1,3,4-thiadiazole (0.2 mol) in ethanol (30 mL) and water (20 mL) at room temperature. The mixture was then refluxed for 6 h.  The reaction mixture was taken up in hexane (100 mL), washed with water and brine, and dried with Na_2_SO_4_. The solvent was removed under reduced pressure, and the crude product was recrystallised from ethanol to give the title compound as colourless solid in 85% yield. Colorless block-like single crystals were obtained by slow evaporation from ethanol at room temperature.

### Refinement

The H atoms were positioned geometrically and treated as riding with d(C-H) = 0.93Å,  U_iso_=1.2U_eq_ (C)

### Crystal data

**Table:**

C_4_H_2_N_4_S_4_	*F*(000) = 472
*M**_r_* = 234.34	*D*_x_ = 1.847 Mg m^−^^3^
Monoclinic, *C*2/*c*	Melting point: 384 K
Hall symbol: -C 2yc	Mo *K*α radiation, λ = 0.71073 Å
*a* = 9.706 (2) Å	Cell parameters from 1881 reflections
*b* = 4.8980 (12) Å	θ = 2.3–28.3°
*c* = 18.008 (5) Å	µ = 1.07 mm^−^^1^
β = 100.074 (3)°	*T* = 291 K
*V* = 842.9 (4) Å^3^	Block, colorless
*Z* = 4	0.29 × 0.20 × 0.11 mm

### Data collection

**Table:**

Bruker SMART CCD area detector diffractometer	783 independent reflections
Radiation source: fine-focus sealed tube	727 reflections with *I* > 2σ(*I*)
graphite	*R*_int_ = 0.017
φ and ω scans	θ_max_ = 25.5°, θ_min_ = 2.3°
Absorption correction: multi-scan (*SADABS*; Bruker, 1997)	*h* = −11→11
*T*_min_ = 0.746, *T*_max_ = 0.889	*k* = −5→5
2915 measured reflections	*l* = −21→21

### Refinement

**Table:**

Refinement on *F*^2^	Primary atom site location: structure-invariant direct methods
Least-squares matrix: full	Secondary atom site location: difference Fourier map
*R*[*F*^2^ > 2σ(*F*^2^)] = 0.021	Hydrogen site location: inferred from neighbouring sites
*wR*(*F*^2^) = 0.055	H-atom parameters constrained
*S* = 1.11	*w* = 1/[σ^2^(*F*_o_^2^) + (0.0269*P*)^2^ + 0.5364*P*] where *P* = (*F*_o_^2^ + 2*F*_c_^2^)/3
783 reflections	(Δ/σ)_max_ = 0.001
55 parameters	Δρ_max_ = 0.17 e Å^−^^3^
0 restraints	Δρ_min_ = −0.26 e Å^−^^3^

### Special details

**Table:**

Geometry. All esds (except the esd in the dihedral angle between two l.s. planes) are estimated using the full covariance matrix. The cell esds are taken into account individually in the estimation of esds in distances, angles and torsion angles; correlations between esds in cell parameters are only used when they are defined by crystal symmetry. An approximate (isotropic) treatment of cell esds is used for estimating esds involving l.s. planes.
Refinement. Refinement of F^2^ against ALL reflections. The weighted R-factor wR and goodness of fit S are based on F^2^, conventional R-factors R are based on F, with F set to zero for negative F^2^. The threshold expression of F^2^ > 2sigma(F^2^) is used only for calculating R-factors(gt) etc. and is not relevant to the choice of reflections for refinement. R-factors based on F^2^ are statistically about twice as large as those based on F, and R- factors based on ALL data will be even larger.

### Fractional atomic coordinates and isotropic or equivalent isotropic displacement parameters (Å^2^)

**Table:**

	*x*	*y*	*z*	*U*_iso_*/*U*_eq_	
S1	0.58334 (4)	0.20034 (8)	0.72250 (2)	0.03599 (15)	
S2	0.37175 (4)	0.60103 (10)	0.63157 (3)	0.04226 (16)	
C1	0.53518 (15)	0.4548 (3)	0.65306 (8)	0.0291 (3)	
C2	0.43894 (19)	0.7877 (4)	0.56554 (9)	0.0393 (4)	
H2	0.3858	0.9153	0.5347	0.047*	
N1	0.62594 (14)	0.5425 (3)	0.61357 (8)	0.0388 (3)	
N2	0.56803 (16)	0.7391 (3)	0.56152 (8)	0.0414 (4)	

### Atomic displacement parameters (Å^2^)

**Table:**

	*U*^11^	*U*^22^	*U*^33^	*U*^12^	*U*^13^	*U*^23^
S1	0.0434 (3)	0.0342 (3)	0.0334 (2)	0.00958 (17)	0.01497 (18)	0.00365 (16)
S2	0.0290 (2)	0.0495 (3)	0.0495 (3)	0.00302 (18)	0.01012 (19)	0.0138 (2)
C1	0.0310 (8)	0.0296 (8)	0.0274 (7)	0.0002 (6)	0.0072 (6)	−0.0023 (6)
C2	0.0433 (10)	0.0400 (10)	0.0333 (9)	−0.0019 (7)	0.0034 (7)	0.0067 (7)
N1	0.0358 (8)	0.0441 (8)	0.0390 (8)	0.0026 (6)	0.0137 (6)	0.0069 (6)
N2	0.0463 (9)	0.0443 (8)	0.0354 (7)	−0.0022 (7)	0.0118 (6)	0.0079 (6)

### Geometric parameters (Å, °)

**Table:**

S1—C1	1.7695 (16)	C1—N1	1.298 (2)
S1—S1^i^	2.0393 (9)	C2—N2	1.290 (2)
S2—C2	1.7164 (17)	C2—H2	0.9300
S2—C1	1.7217 (16)	N1—N2	1.392 (2)
			
C1—S1—S1^i^	102.08 (5)	N2—C2—S2	115.54 (13)
C2—S2—C1	86.01 (8)	N2—C2—H2	122.2
N1—C1—S2	115.22 (12)	S2—C2—H2	122.2
N1—C1—S1	119.96 (12)	C1—N1—N2	111.40 (13)
S2—C1—S1	124.81 (9)	C2—N2—N1	111.82 (14)
			
C2—S2—C1—N1	−0.11 (13)	S2—C1—N1—N2	−0.16 (18)
C2—S2—C1—S1	−179.53 (11)	S1—C1—N1—N2	179.30 (11)
S1^i^—S1—C1—N1	169.70 (12)	S2—C2—N2—N1	−0.5 (2)
S1^i^—S1—C1—S2	−10.90 (11)	C1—N1—N2—C2	0.4 (2)
C1—S2—C2—N2	0.38 (15)		

Symmetry codes: (i) −*x*+1, *y*, −*z*+3/2.

### Hydrogen-bond geometry (Å, °)

**Table:**

*D*—H···*A*	*D*—H	H···*A*	*D*···*A*	*D*—H···*A*
C2—H2···N2^ii^	0.93	2.52	3.249 (2)	136

Symmetry codes: (ii) −*x*+1, −*y*+2, −*z*+1.
